# Definitive IMRT for Stage III Thymic Carcinoma: A Brief Report and Literature Review

**DOI:** 10.3389/fonc.2016.00219

**Published:** 2016-10-24

**Authors:** Sarah A. Dooley, Carryn M. Anderson

**Affiliations:** ^1^Carver College of Medicine, University of Iowa, Iowa City, IA, USA; ^2^Department of Radiation Oncology, University of Iowa Hospital and Clinics, Iowa City, IA, USA

**Keywords:** thymic carcinoma, definitive IMRT, thoracic cancer, radiation therapy, chemotherapy

## Abstract

**Introduction:**

Thymic carcinoma is a rare malignancy often presenting at an advanced stage. Radiation therapy and chemotherapy are often the only treatment options available to physicians.

**Methods:**

A 70-year-old man presented with an unresectable stage III thymic tumor and was treated with 45 Gy in 25 fractions followed by a boost of 21.6 Gy in 12 fractions. He was also treated with bortezomib for multiple myeloma unrelated to his primary malignancy.

**Results:**

The patient made a full recovery following the radiation regimen and remained disease free 4 years after the treatment.

**Conclusion:**

Exclusive treatment with intensity-modulated radiation therapy provides a viable treatment option for patients presenting with advanced stage thymic carcinoma.

## Introduction

Thymoma and thymic carcinoma are primary tumors of the thymus that arise in the anterior mediastinum. Thymomas are histologically benign and are capable of behaving in a malignant fashion by extending into the thymic capsule (1). On the one hand, thymomas have a reported incidence of 1.5 cases per million (2). On the other hand, thymic carcinomas are even more rare in the population and are histologically malignant. They tend to demonstrate a more aggressive behavior by invading the thymic capsule early on. Given the rarity of thymic carcinoma, studies of thymic carcinoma are predominantly retrospective case reports spanning a number of decades at a single institution. The risk factors for thymic carcinoma remain unknown; however, thymic masses generally occur in adults (3). Treatment options include surgical resection, chemotherapy, and/or radiotherapy with complete surgical resection having the most favorable outcome (4–6). Tumor grade, stage, and resectability are essential prognostic factors (7).

Surgical resection of thymic carcinoma is not always an option as the majority of thymic tumors present at an advanced stage. Other medical comorbidities may also play a role in whether a patient undergoes surgery. In these circumstances, chemotherapy and/or radiation therapy can be used. We include a case of a 70-year-old man diagnosed with stage III thymic carcinoma managed with definitive radiation therapy alone and discuss literature as it relates to radiation as a curative modality for this disease.

## Case Presentation

**Figure 1:**
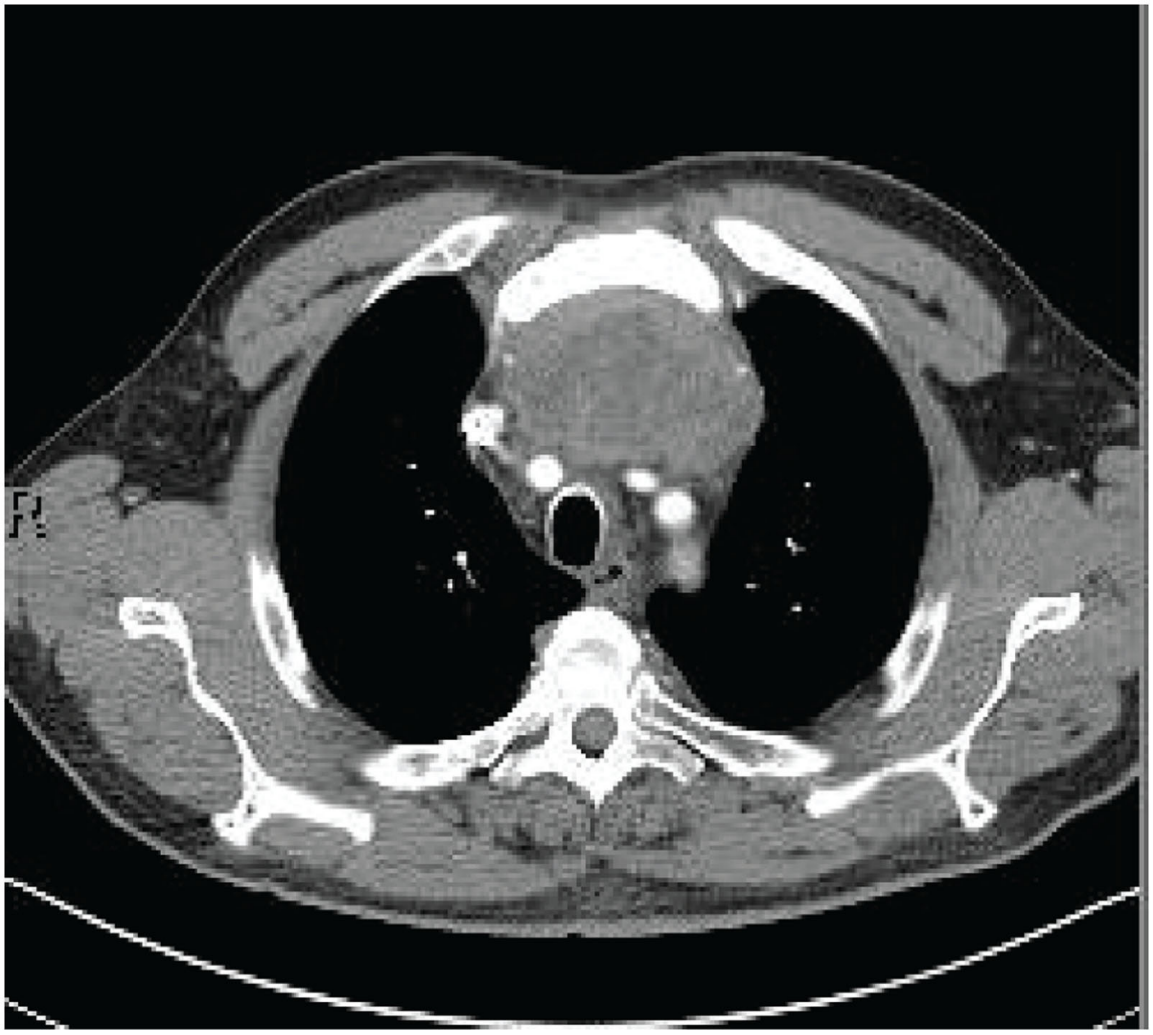
**CT scan prior to radiation**.

**Figure 2:**
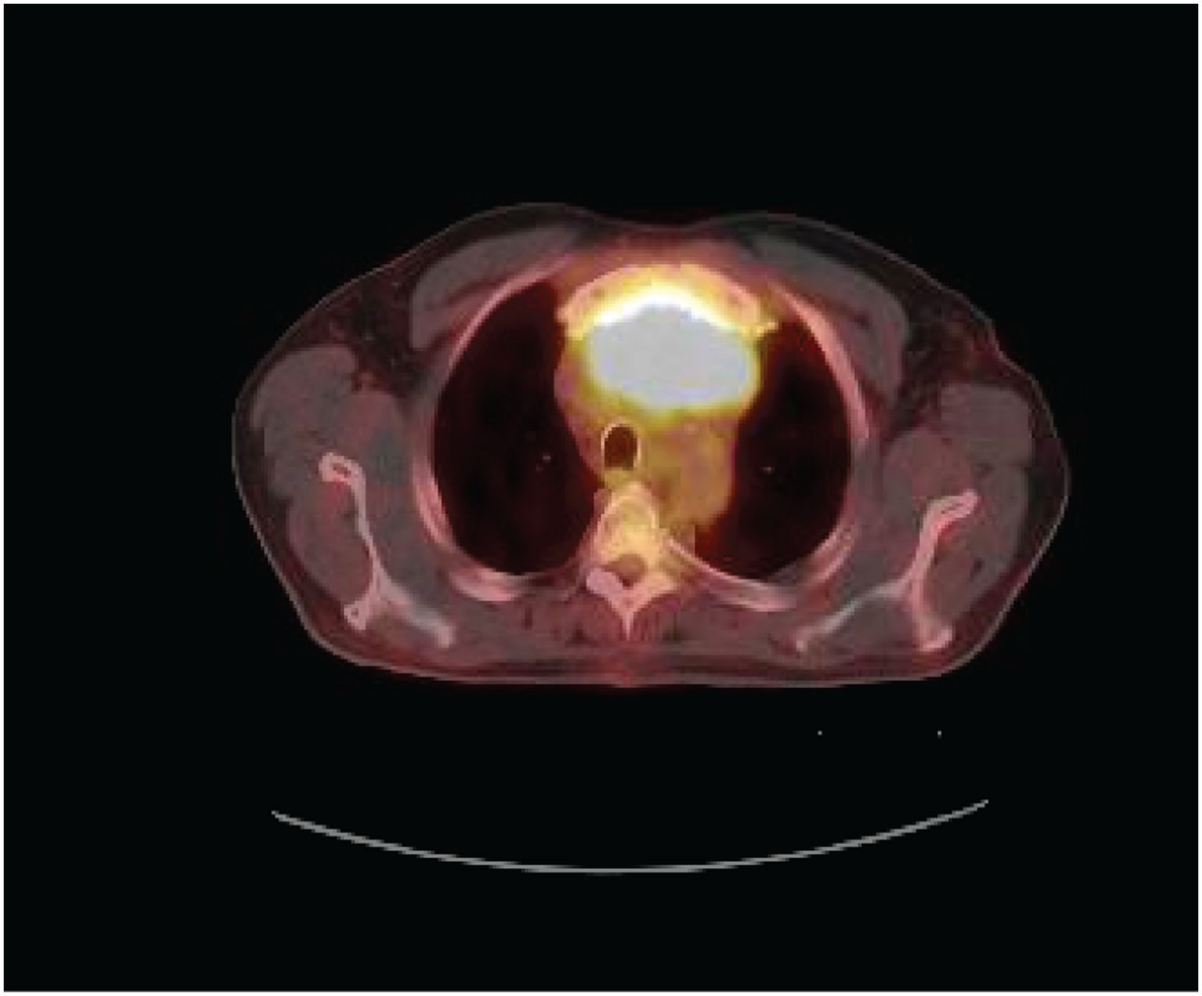
**PET scan prior to radiation**.

A 70-year-old man presented to his primary physician with dysphagia that progressed to upper chest and back-pressure. The patient underwent imaging, and a large anterior mediastinal mass was detected on computed tomography (CT). The mass was biopsied, and the patient was subsequently referred for further work-up. Pathology of the tumor was reviewed and confirmed to be poorly differentiated squamous cell carcinoma. The tumor histology was most consistent with thymic origin; however, metastasis of other sources, such as lung, could not be ruled out. A repeat CT scan (Figure 1) showed a necrotic mediastinal mass with local sternal invasion. An initial positron emission tomography (PET) scan (Figure 2) showed a hypermetabolic anterior mediastinal mass without evidence of regional or distant metastases. The anterior mediastinal location made the mass most likely of thymic source. Upon presentation, the patient denied any hemoptysis, voice changes, fatigue, weakness, vision changes, headaches, nausea, or vomiting. The patient underwent surgery for removal of the tumor. During the operation, the tumor was found to invade the sternum and bilaterally encase both the phrenic nerves. In attempts to see if the phrenic nerves could be freed without injury, the pericardial sac was opened and the right phrenic nerve was followed. Based on the extent of tumor invasion of the right phrenic nerve, the surgeon concluded that there was no safe way to free both the phrenic nerves without injury, and the tumor was concluded to be unresectable.

**Figure 3:**
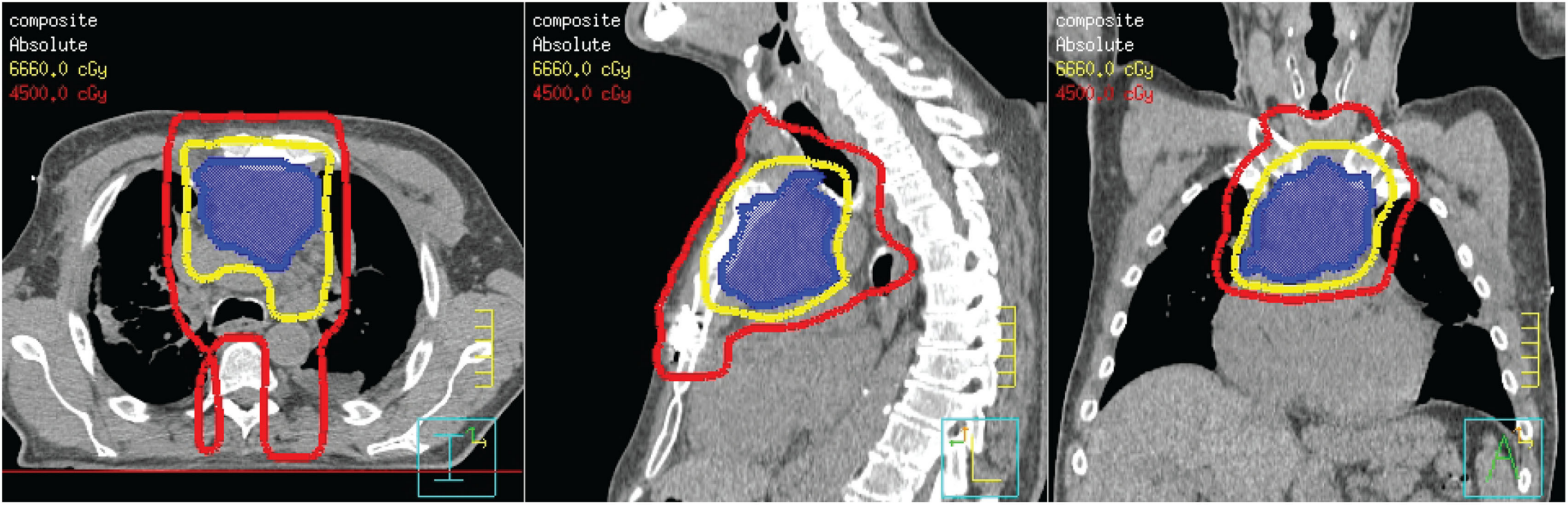
**Radiation treatment plan**.

The patient was referred to oncology and radiation oncology for other definitive therapy options. The patient was not considered a candidate for cisplatin therapy due to a rising protein–creatinine ratio at the time of presentation. A biopsy obtained for further work-up of his poor kidney function showed lambda light chain deposits consistent with multiple myeloma. After consultation with radiation oncology, intensity-modulated radiation therapy (IMRT) was recommended for definitive management of his thymic carcinoma. The plan was designed to deliver volumes based on the image data seen in Figure 3. A dose of 45 Gy in 25 fractions (red line) was prescribed to cover the thoracotomy operative bed, mediastinum, and level VI lymph node region of the low neck. A boost plan of 21.6 Gy in 12 fractions was designed to cover the gross tumor (blue color wash), bringing the total dose to the gross tumor to 66.6 Gy (yellow line). IMRT resulted in a lower dose to the spinal cord and heart while maintaining target coverage compared to a 3D-conformal technique. In addition, the patient was started on a regimen of bortezomib and dexamethasone for treatment of multiple myeloma during this time.

Overall, the patient tolerated the treatment well and did not experience any significant side effects other than skin irritation. The patient did notice gradual improvement in his dysphagia throughout treatment. A CT performed 1 month following the completion of his treatment showed a decrease in the size of his mediastinal mass (6.8 cm × 3.0 cm × 4.2 cm) when compared to the mass initial size prior to radiation (8.4 cm × 5.7 cm × 7.8 cm). The CT also showed no enlarged lymph nodes of the axilla or mediastinum and was negative for pulmonary metastases. Imaging revealed a new right pericardial thickening that was later determined to be radiation-induced inflammation. The patient had significant improvement in kidney function after completing 22 cycles of bortezomib and dexamethasone for his multiple myeloma. His protein–creatinine ratio normalized within 6 months of this treatment. A bone marrow biopsy prior to treatment showed 30% plasma cells and 1% plasma cells following treatment. He continues to stay on a maintenance dose of dexamethasone.

The patient was last seen in radiation oncology 4 years following the completion of his radiation regimen. He reports that he is doing well and is symptom free. To date, the patient’s CT scan continues to show a stable small soft tissue mass in the anterior mediastinum with surrounding fat stranding consistent with radiation change.

## Discussion

Due to the rarity of thymic carcinoma, large prospective studies on the therapeutic management of thymic carcinoma are limited. As a result, clinical management of thymic carcinoma is poorly defined. Radiation therapy alone for the definitive treatment of thymic carcinoma is not well reported as these cases are often grouped together with thymomas. The National Comprehensive Cancer Network (NCCN) recommends a radiation dose range of 60–70 Gy for inoperable tumors. For operable tumors undergoing adjuvant radiotherapy, the NCCN specifies 45–50 Gy for clear margins, 54 Gy for microscopically positive margins, and 60 Gy for positive gross margins post resection (2). The range 1.8–2.0 Gy is considered a conventional fractionation (2).

Using radiation therapy as a definitive treatment has been suggested in a number of studies. In Korst et al., 21 patients with thymic carcinoma and thymoma underwent neoadjuvant chemoradiotherapy (2 cycles of etoposide and cisplatin) and 45 Gy of radiotherapy. Eighteen of the 19 patients, who had reported PET SUVmax values, experienced a decrease in their SUVmax with the median change of −44.5%. The study also observed a greater tumor response in thymic carcinoma lesions in comparison to thymoma lesions. Four of the five patients who had obtained near to complete pathologic response were patients with thymic carcinoma (8).

Studies have suggested the benefits of radiation therapy when used with neoadjuvant and adjuvant therapy by making tumors more resectable and improving local control, respectively (9). Hsu et al. showed promising local control in thymic carcinoma tumors that underwent complete or incomplete surgical resection with 40–70 Gy of radiotherapy. The 5-year local control rate in completely and incompletely resected thymic carcinoma tumors plus radiotherapy was 92 and 88%, respectively. The study concluded that the local control was notable; however, the average distant metastasis-free rate of 57% was unimpressive (10). Overall, the study showed the benefit of radiation therapy in incompletely resected tumors which is comparable to inoperable tumors. In the study of Liu et al., 17 patients with unresectable thymic malignancies who underwent radiotherapy and/or chemotherapy were compared to 4 patients with unresectable thymic tumors who did not undergo treatment. Although the study presented a small cohort, it is worth noting as it is one of the few papers to show benefit in treatment versus no treatment in inoperable tumors (7).

The use of proton therapy has been explored in the treatment of mediastinal masses. Although systematic reviews suggest that proton therapy has not proven to be superior to photon therapy, more recent studies have shown the potential of less toxicity in lung cancer patients receiving proton therapy (11). Chang et al. showed that 34 patients with lung and mediastinal tumors who received a median of 66 Gy of intensity-modulated proton therapy (IMPT) had significantly lower radiation dosage to normal tissues (heart, lung, and esophagus), when compared to IMRT plans that were contoured for the same patients’ tumors (12). More clinical trials are needed to further explore the possible improvement in the side effect profile proton therapy could offer.

In this case, the patient’s thymic carcinoma has been found to be unresectable due to its involvement with both the phrenic nerves. Chemotherapy and radiation therapy were both explored as alternative definitive treatment options. The patient’s renal function made him a poor candidate for the chemotherapy regimen of choice, cisplatin. He was a good candidate for radiation therapy and successfully completed a regimen of 66.6 Gy/37 fractions. It is important to note that the patient started bortezomib for multiple myeloma during his radiation therapy and continued bortezomib post-radiation. It is uncertain whether bortezomib played a role in the patient’s treatment outcomes. Overall, the reduction in the patient’s tumor size and persistent local control suggest that IMRT is a useful definitive treatment option for unresectable thymic carcinoma tumors.

## Ethics Statement

This study was carried out in accordance with the appropriate federal regulations as well as in accordance with the radiation oncology registry approved by the University of Iowa Institutional Review Board (IRB 01, approval 201109821). Written and informed consent was obtained from participants as per IRB recommendations. This registry does not meet the WHO definition of a clinical trial and is considered exempt from clinicaltrials.gov requirements.

## Author Contributions

SD is the first author, and CA is the corresponding author. SD drafted the manuscript, and CA provided critical revision. Both authors contributed to the design, analysis, and interpretation of this case study. Both authors approved the final version to be submitted for publication, and both remain accountable for the integrity of the study.

## Conflict of Interest Statement

The authors declare that the research was conducted in the absence of any commercial or financial relationships that could be construed as a potential conflict of interest.
